# The incidence of postural orthostatic tachycardia syndrome in the population of Zagreb, Croatia

**DOI:** 10.3325/cmj.2020.61.422

**Published:** 2020-10

**Authors:** Ivan Adamec, Luka Crnošija, Berislav Ruška, Tin Pavičić, Anamari Junaković, Magdalena Krbot Skorić, Tatjana Pekmezović, Mario Habek

**Affiliations:** 1 Department of Neurology, Referral Center for Autonomic Nervous System Disorders, University Hospital Center Zagreb, Zagreb, Croatia; 2 Faculty of Electrical Engineering and Computing, University of Zagreb, Zagreb, Croatia; 3 Institute of Epidemiology, Faculty of Medicine, University of Belgrade, Belgrade, Serbia; 4 University of Zagreb School of Medicine, Zagreb, Croatia

## Abstract

**Aim:**

To estimate the incidence of postural orthostatic tachycardia syndrome (POTS) in the population of Zagreb, Croatia, and to determine the patients’ demographic and clinical characteristics.

**Methods:**

From 2012-2017, we identified patients with POTS by a retrospective analysis of medical records at University Hospital Center Zagreb. Crude incidence rates were directly standardized by age according to the European and World Standard Population.

**Results:**

Out of 385 patients with suspected POTS, 23 had a definitive POTS diagnosis. The annual incidence ranged from 3.3 to 14.8 per 1 000 000 for both sexes combined. The highest incidence rates were in the age groups 18-29 and 30-39 years, with female predominance. The mean age at diagnosis was 30.7 years (standard deviation ±9.2, range 18-52). The median duration of symptoms at diagnosis was 7.5 months (range 3-180 months). Regarding associated comorbidities, two patients had chronic gastritis and one patient had each of the following: epilepsy, prior subarachnoid hemorrhage, anxiety, mitral insufficiency, obstructive sleep apnea, hypothyreosis, and irritable bowel syndrome. In patients not fulfilling the criteria for POTS, the most common alternative diagnoses were autonomic dysfunction due to multiple sclerosis in 22, anxiety disorder in 17, epilepsy in 16, and orthostatic tachycardia due to deconditioning in 13 patients.

**Conclusion:**

The data obtained in this study can be used to optimize disease surveillance in population, comprehensive assessment of disease burden, and organization of health care services.

Postural orthostatic tachycardia syndrome (POTS) is a functional disorder of the autonomic nervous system (ANS). It is defined as a sustained increase in heart rate of over 30 beats per minute in the upright position without orthostatic hypotension. In order for the diagnosis to be made, typical orthostatic symptoms have to last for more than three months (1). POTS occurs mostly in young individuals, more frequently female, with 25% of patients having a positive family history (2). Typical symptoms include lightheadedness, palpitations, and general malaise when being upright, with low tolerance of physical exertion. Other autonomic symptoms, beside cardiovascular, can be present, such as gastric and sudomotor symptoms (3). Headaches and sleeping difficulties are commonly present, and POTS occurrence seems to be associated with joint hypermobility (4,5). One of the most troublesome symptoms is the occurrence of “brain fog,” a cognitive dysfunction characterized by difficulty focusing and thinking, leading to poor intellectual performance (6). The substantial burden of POTS symptoms leads to impaired physical and social functioning (7), and, in a quarter of patients, impaired work ability (8). While the prognosis is generally favorable, as much as 40% of those affected are not able to achieve the levels of functioning they had before the diagnosis (9). The underlying pathophysiological mechanisms leading to POTS are not completely understood. However, it seems that there are two main types of POTS, the adrenergic and neuropathic type (10). The adrenergic type is characterized by high plasma norepinephrine (NE) levels due to NE synaptic spillover, with values in the upright position exceeding 3.5 nmol/L. The neuropathic type is characterized by limited lower limb sympathetic denervation (2). Two other major contributors to the development of POTS are hypovolemia and deconditioning (11).

Although POTS has been well characterized, the disease burden is still not completely known. The main reasons for this are heterogeneous clinical presentation and lack of knowledge on this condition. This is emphasized by a study showing that two thirds of people with POTS report at least ten different symptoms, making the diagnostic process cumbersome (12). Similarly, another study showed that people with POTS waited approximately 4 years from presentation to a correct diagnosis and were often suspected of having a psychiatric condition (13). All this indicates the lack of epidemiological data on POTS. The prevalence is often estimated at 1 in 10 000, although exact data supporting this number are missing (11,14,15). One example of such estimation is a recent review by Arnold et al from 2018 reporting a prevalence of 0.1 to 1% in the United States population. The article references two publications, one presenting the outcomes of adolescent-onset POTS and the other being an editorial (11,14,15). Another example is a review by Matthias et al, which estimated a prevalence of 170 cases per 100 000, referencing an article on orthostatic intolerance in a group of patients with chronic fatigue syndrome (16,17). The only data on POTS incidence are available from the study by AbdelRazek et al, who reported an incidence of 6 per 100 000 in Olmsted County, USA, in 2016 (18). Therefore, further studies are needed to more accurately assess the epidemiology and demographic characteristics of POTS patients. The aim of this study was to estimate the incidence of POTS in the population of Zagreb, Croatia, and determine the patients' demographic and clinical characteristics.

## Patients and methods

This retrospective study was conducted in University Hospital Center Zagreb, Croatia. The Referral Center for the Autonomic Nervous System disorders is the only center in Croatia where the diagnosis of ANS abnormalities can be confirmed, and where all patients with suspected ANS diseases from Croatia are referred to. For the purpose of this study, all three other neurological departments in Zagreb were contacted, confirming that all suspected POTS patients in the studied period were referred to the Referral Center for confirmation of the diagnosis.

**Figure 1:**
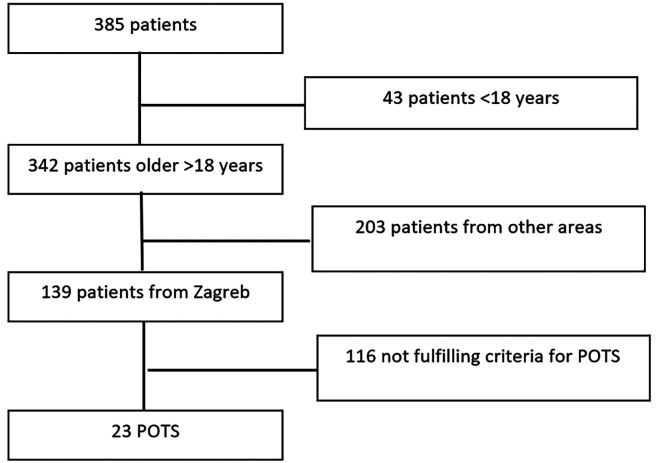
The flowchart of the study. POTS – postural orthostatic tachycardia syndrome.

All patients referred from 2012 to 2017 with a suspected POTS diagnosis and with regular follow-up in the outpatient clinic were eligible for inclusion (Figure 1). After the extraction of all patients' data, only patients older than 18 years and those residing in the City of Zagreb were considered for further analysis. The following data were collected: age, sex, duration of symptoms, supine and standing NE levels, and comorbidities.

Three investigators (MH, IA, and LC) independently reviewed medical records of all 139 patients fulfilling the inclusion criteria in order to confirm the diagnosis of POTS. The diagnosis of POTS was based on the following criteria:

1. Sustained increment of heart rate (HR)≥30 bpm or an average HR of ≥120 bpm in the absence of orthostatic hypotension on tilt table test (1);

2. A history of orthostatic intolerance symptoms lasting at least 3 months (19);

3. In order to check for possible deconditioning, it had to be confirmed that patients did not suffer from systemic illnesses or other medical issues leading to prolonged bed rest.

Patients were included in the final analysis if two out of three investigators confirmed the diagnosis of POTS. The main reason for this approach was that POTS patients typically present with a myriad of symptoms, so in some cases the diagnosis can be doubtful.

The population data of the Zagreb administrative region for each of the studied years was obtained from the Croatian Bureau of Statistics in the form of population projections based on the 2011 census (20). The study was approved by the University Hospital Center Zagreb Ethics Committee.

### Statistical analysis

The incidence rates are provided in 10-year age groups, ranging from 18 up to ≥70 years, and for a 6-year time period (2012-2017), and were calculated according to the date of POTS diagnosis. The 95% confidence intervals (CI) for incidence rates are based on Poisson frequency distribution for rarely occurring events (21). All crude incidence rates were directly standardized by age according to the European Standard Population (22) and World Standard Population (23) in order to eliminate the influence of different age structures in the Croatian population.

## Results

### The incidence of POTS

From 2012 to 2017, 385 patients with a suspected POTS diagnosis were referred to the Referral Center for the Autonomic Nervous System disorders. Out of these, at the time of examination 342 patients were older than 18 years, and 139 had permanent residence in the City of Zagreb. The final analysis included 23 patients from this group in whom the diagnosis of POTS was confirmed by two out of three investigators. All three investigators confirmed the diagnosis in 16 patients, and two out of three investigators confirmed it in 7 patients. The only reason for disagreement between the investigators was the POTS criterion number 2 (“A history of orthostatic intolerance symptoms lasting at least 3 months”). In the group of patients without confirmed POTS diagnosis (116 patients), for only 14 patients one of the investigators confirmed the diagnosis.

In the confirmed POTS group, there were 18 (78.3%) female patients, with female to male ratio of 3.6:1. The mean age at time of diagnosis was 30.7 (SD±9.2), with the youngest patient being 18 and oldest 52 years of age. The median duration of symptoms at diagnosis was 7.50 months (range 3 to 180 months) (Table 1). The annual POTS incidence ranged from 3.3 to 14.8 per 1 000 000 for both sexes combined, with higher values among women (Table 2).

**Table 1:** Age and duration of symptoms of postural orthostatic tachycardia syndrome patients included in the study

	Mean	Median	Standard deviation	Minimum	Maximum
Age (years)	30.67	31.72	9.15	18.28	52.79
Duration of symptoms (months)	17.50	7.50	37.17	3.00	180.00
Norepinephrine supine (nmol/L)	1.65	1.54	0.77	0.62	2.99
Norepinephrine standing (nmol/L)	3.38	3.11	1.42	2.02	6.21

**Table 2:** Annual crude incidence rates of postural orthostatic tachycardia syndrome in the period 2012–2017

Year	2012	2013	2014	2015	2016	2017
Number of cases						
male	0	1	2	0	2	0
female	2	1	4	2	7	2
both sexes	2	2	6	2	9	2
Population						
male	279 803	280 199	280 544	280 666	281 728	281 896
female	323 237	323 696	324 083	324 120	325 012	324 980
both sexes	603 040	603 895	604 627	604 786	606 740	606 876
Incidence rate (/1 000 000)						
male	0.0	3.6	7.1	0.0	7.1	0.0
female	6.2	3.1	12.3	6.2	21.5	6.2
both sexes	3.3	3.3	9.9	3.3	14.8	3.3
95% confidence interval						
male	0.0-0.0	0.1-20.1	0.9-25.6	0.0-0.0	0.9-25.6	0.0-0.0
female	0.8-22.4	0.1-17.3	3.3-31.5	0.8-22.4	8.6-44.3	0.8-22.4
both sexes	0.4-11.9	0.4-11.9	3.6-21.5	0.4-11.9	7.1-28.1	0.4-11.9

The incidence rates of POTS adjusted to the European Standard Population were 6.4/1 000 000 for both sexes: 9.4/1 000 000 for women and 2.6/1 000 000 for men (Table 3). When we used World Standard Population, lower incidence rates were obtained: 7.5/1 000 000 for women and 2.1/1 000 000 for men, and 5.1/1 000 000 for both sexes.

**Table 3:** Average annual crude and standardized incidence rates of postural orthostatic tachycardia syndrome in the period 2012-2017

		Incidence rate (1 000 000) standardized to	
	Crude incidence rate (1 000 000)	European Standard Population	World Standard Population
Male	3.0	2.6	2.1
Female	9.3	9.4	7.5
Both sexes	6.3	6.4	5.1

The highest age-specific incidence rates in both sexes were observed in the age groups 18-29 and 30-39 years, while in the age groups older than 60 years no POTS cases were observed (Table 4).

**Table 4:** Average annual age- and sex-specific incidence rates of postural orthostatic tachycardia syndrome in the period 2012-2017

Sex	Age groups	Incidence rate (/1 000 000)	95% confidence interval
Male	18-29	7.0	2.8-14.4
	30-39	8.1	3.5-15.8
	40-49	0.0	0.0-0.0
	50-59	0.0	0.0-0.0
	60-69	0.0	0.0-0.0
	70+	0.0	0.0-0.0
Female	18-29	27.3	16.7-42.2
	30-39	20.5	15.5-31.7
	40-49	2.9	0.6-8.5
	50-59	2.9	0.6-8.5
	60-69	0.0	0.0-0.0
	70+	0.0	0.0-0.0
Both	18-29	17.3	10.1-27.7
	30-39	17.2	10.0-27.5
	40-49	1.5	0.2-5.4
	50-59	1.6	0.2-5.8
	60-69	0.0	0.0-0.0
	70+	0.0	0.0-0.0

### Type of POTS

Norepinephrine values in the supine position and after 10 min of passive tilting to 70° were available for 16 patients. The mean NE values in the supine and tilted position are presented in Table 1. Five (31.3%) patients had upright NE levels above the threshold of 3.5 nmol/L.

### POTS comorbidities

Comorbidities were assessed from the electronic records for all 23 patients diagnosed with POTS. There were two patients with chronic gastritis. The following comorbidities were identified in one patient each: epilepsy, prior subarachnoid hemorrhage, anxiety, mitral valve insufficiency, obstructive sleep apnea, hypothyreosis, and irritable bowel syndrome.

### Alternative diagnoses in patients referred to as suspected POTS

In 116 patients who were not diagnosed with POTS, the most common diagnosis was autonomic dysfunction due to multiple sclerosis, which was made in 22 patients. Seventeen patients had anxiety disorder, 16 patients had epilepsy, and 13 patients had orthostatic tachycardia due to deconditioning. Ten patients were shown to have orthostatic hypotension during tilt table testing and 10 more patients did not have an increase over 30 beats per minute in the upright position, thus not fulfilling the criteria for POTS (Table 5).

**Table 5:** Alternative diagnosis in patients with suspected postural orthostatic tachycardia syndrome

Diagnosis	N = 116
Multiple sclerosis	22
Anxious disorder	17
Epilepsy	16
Deconditioning	13
Anemia	11
Orthostatic hypotension	10
Heart rate increase <30/min	10
Syncope	9
Hyperthyreosis	3
Polyneuropathy	2
Supraventricular tachycardia	2
Enterovirosis	1

## Discussion

The results of the current study show an incidence of POTS in Zagreb ranging from 3.3 to 14.8 per 1 000 000, with average annual value of 6.3/1 000 000. These rates are lower than the incidence of 6 per 100 000 reported by AbdelRazek et al in the population of Olmstead County, Minnesota, USA (18). The difference could be attributed to geographical and genetical variations or a different effect of environmental factors. The etiology of POTS is not completely understood, but about 50% of patients reported viral infections preceding the symptoms and 25% of patients reported a positive family history (2). AbdelRazek et al have observed an increase in POTS incidence from 2000 to 2016, which may reflect an increased awareness and recognition of the syndrome among patients and physicians (18). Similarly, researchers in Denmark have noticed an increase in the frequency of POTS diagnosis made by tilt-table testing, which was actually preceded by an increase in the number of POTS-related articles in PubMed (24). Therefore, the lower incidence observed in the current study might reflect a lower awareness of POTS in this particular part of Europe.

The crude incidence comparison can be confounded by differences in the underlying population age structure, which may lead to an inaccurate interpretation of the disease risk (25). In order to allow comparisons of the incidence rates related to different population structures, we used the European and world population as a standard, an approach widely applied in many epidemiological investigations.

The mean age at POTS diagnosis of 30.7 years and the female predominance (female to male ratio of 3.6:1) observed in our study are in line with previous findings (11). In the current study, NE levels in the supine and tilted position were available for 16 patients, with five (31.3%) patients having an upright level of NE above the threshold of 3.5 nmol/L, indicating an increased sympathetic drive. Between a third and a half of all patients with POTS belong to the hyperadrenergic type, characterized by high standing plasma NE levels, and this study supports these findings (2). The increase in NE may be caused by a reduced clearance of synaptic NE due to the NE transporter deficiency, however this pathophysiological mechanism has been genetically confirmed in only one family (26). In the neuropathic POTS, rather than expressing high NE levels in the upright position, patients are thought to experience venous pooling in the legs while standing due to impaired peripheral vasoconstriction (2). In the current study, 69.7% of patients had NE levels lower than 3.5 nmol/L in the tilted position. However, in this group of patients we did not systematically perform ancillary studies, such as quantitative sudomotor axon reflex testing, which prevented us from confirming sympathetic denervation of the lower limbs. It is important to stress that POTS classification into two subtypes is somewhat artificial as these two mechanisms can overlap, and POTS occurrence is further influenced by additional factors, such as hypovolemia and deconditioning (27). Nevertheless, the findings of this study corroborate the fact that there is a subset of POTS patients with an increased orthostatic sympathetic activity.

POTS has been associated with various comorbidities, such as Ehlers-Danlos syndrome, chronic fatigue syndrome, or irritable bowel syndrome, and these comorbidities may in fact represent a wider spectrum of the syndrome itself (27). However, out of these comorbidities, in the current study only irritable bowel syndrome was recognized in one patient. This may be due to the study’s retrospective nature and the comorbidities being extracted from the electronic records. As the presence of symptoms such as joint hypermobility or chronic fatigue was not actively investigated, they might have been missed. We observed no apparent clustering of comorbidities, with two patients experiencing chronic gastritis and no more than one patient experiencing all the other comorbidities.

Out of 139 patients from Zagreb who were suspected of having POTS, 116 did not fulfill the criteria. The most common alternative diagnoses were autonomic dysfunction due to multiple sclerosis, anxious disorder, and epilepsy. The prevalence of these diagnoses can be explained by the fact that the center where the current study was performed receives frequent referrals for autonomic function testing in multiple sclerosis and epilepsy patients. Anxiety is not uncommon in POTS patients and can exacerbate the reported symptoms (28). On the other hand, symptoms such as palpitations, sweating, and tremor can in fact be a manifestation of an anxiety disorder, and this has to be taken into account when dealing with patients with suspected orthostatic intolerance (29). After performing tilt table testing on a group of suspected POTS patients in this study, ten were found to have orthostatic hypotension and nine had syncope. In patients with orthostatic symptoms, tilt table testing is the preferred tool for differentiating between these types of orthostatic intolerance. Patients with orthostatic hypotension, as well as those with POTS, experience their symptoms during the upright position, with relief when they change their position to supine. Both groups of patients experience postural symptoms, such as general malaise, lightheadedness, and blurred vision (30). Furthermore, syncope is experienced by about 30% of patients with POTS (27). Therefore, for establishing the correct diagnosis in patients with symptoms of poor orthostatic tolerance it is essential to perform autonomic testing.

The main limitations of this study are referral bias as the study was performed in the referral center and a relatively small number of patients. Since the study was conducted retrospectively, data such as specific comorbidities related to POTS might have been missed and therefore underrepresented in this population, and the small sample size may not have uncovered some of the other associated diseases. Nevertheless, this study presents a valuable addition to the currently scarce and insufficient epidemiological data on POTS.

In conclusion, in order to better estimate the risk of POTS, data obtained in this study can be used in optimizing surveillance of the disease in population, comprehensive assessment of disease burden, and organization of health care services. Further studies are necessary to explore the incidence in different populations to estimate time and space variations in disease frequency.
